# Identification of Candidate Blood Biomarkers of Recombinant Human Erythropoietin Administration Using Targeted Polar Metabolomics by HILIC‐MS/MS

**DOI:** 10.1002/dta.3943

**Published:** 2025-08-31

**Authors:** Olivier Salamin, Lejla Ramic, Raul Nicoli, Serge Rudaz, Davy Guillarme, Tiia Kuuranne

**Affiliations:** ^1^ Swiss Laboratory for Doping Analyses, University Center of Legal Medicine, Lausanne and Geneva Lausanne University Hospital and University of Lausanne Lausanne Switzerland; ^2^ School of Pharmaceutical Sciences University of Geneva Geneva Switzerland; ^3^ Institute of Pharmaceutical Sciences of Western Switzerland University of Geneva Geneva Switzerland

## Abstract

Increasing oxygen transport through elevated hemoglobin concentration and red blood cell mass is a key objective of blood doping, commonly achieved via recombinant human erythropoietin (rHuEPO) administration or blood transfusions. While the Athlete Biological Passport (ABP) offers an effective indirect tool for detecting such manipulations, its sensitivity and specificity may be limited, particularly in cases involving microdoses or confounding physiological factors. To address these limitations, the identification of novel biomarkers that complement current ABP markers is essential.

This study presents a targeted metabolomics approach to discover candidate biomarkers of rHuEPO administration by analyzing polar metabolites in plasma and serum from two administration studies: one involving a single CERA injection, and the other using multiple doses of epoetin delta. Hydrophilic interaction chromatography hyphenated with tandem mass spectrometry enabled the selective and sensitive detection of a panel of polar endogenous metabolites.

Following data normalization and stringent quality control, generalized least squares models were applied to evidence temporal changes in metabolite signals. Among the most responsive and concordant markers across both studies were hypoxanthine and inosine, which showed significant and marked increases following rHuEPO administration. Notably, the relative increase of these metabolites coincided with the maximum in reticulocyte percentages, reflecting maximal erythropoietic activity. As intermediates in purine metabolism, their increases are likely tied to augmented purine turnover during red blood cell production. These findings suggest that hypoxanthine and inosine are promising candidate biomarkers to complement existing ABP parameters. However, further validation is required to confirm their reliability and applicability within the ABP framework.

## Graphical abstract

Using targeted HILIC‐MS/MS metabolomics, inosine and hypoxanthine were identified as potential biomarkers of EPO administration. Their consistent and sensitive plasma responses highlight their promise for inclusion in the Athlete Biological Passport (ABP), warranting further investigation to enhance anti‐doping detection strategies.

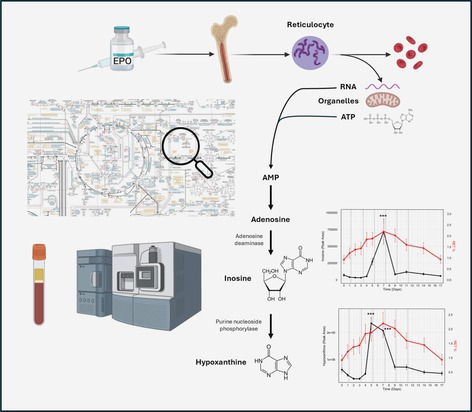

## 1 Introduction

The amount of hemoglobin and circulating red blood cells represents a critical determinant of aerobic performance, as these parameters directly influence the oxygen‐carrying capacity of the organism. Enhancing this capacity is the primary goal of blood doping, a practice aimed at improving endurance and overall athletic performance. Blood doping strategies typically involve either the administration of recombinant human erythropoietin (rHuEPO) or blood transfusions, which may be autologous (using the athlete's own stored blood) or homologous (using blood from a donor).

Direct detection methods are available for both rHuEPO and homologous blood transfusions. These analyses rely on sophisticated techniques, such as sarcosyl‐polyacrylamide gel electrophoresis (SAR‐PAGE) and western blotting to detect the different rHuEPOs, or flow cytometry for identifying mixed red blood cell populations in homologous transfusions [1, 2, 3, 4]. However, such analyses are not systematically applied to every doping control sample. Instead, they are performed upon specific request, typically when there is reasonable suspicion of blood doping or based on findings from indirect monitoring tools like the hematological module of the Athlete Biological Passport (ABP). The latter serves as a critical tool in anti‐doping efforts by indirectly detecting blood doping. It monitors changes in an athlete's hematological parameters, such as hemoglobin concentration, reticulocyte percentage, and other blood markers, to identify abnormalities inconsistent with natural physiology and the athlete's own longitudinal baseline [5, 6, 7]. These deviations may suggest the use of rHuEPO, blood transfusions, or other erythropoiesis‐enhancing methods. In some cases, the ABP alone can provide sufficient evidence of anti‐doping rule violation. Additionally, ABP findings can be used to target further analyses, such as applying direct detection methods for rHuEPO or homologous blood transfusions, enhancing the likelihood of uncovering doping practices.

While the ABP has already proven to be a powerful deterrent and an effective tool for detecting blood doping, some limitations remain, particularly in cases involving low doses of rHuEPO and/or microtransfusions. These approaches can alter hemoglobin and reticulocyte values, while keeping them within the range of natural variability, thereby evading detection. Furthermore, confounding factors such as altitude exposure, which stimulates erythropoiesis, can cause difficulties in the interpretation of an athlete's profile and mask the effects of rHuEPO administration.

In response to these challenges, recent research has focused on identifying additional and complementary biomarkers to enhance the sensitivity and specificity of the ABP [8, 9]. Biomarkers related to iron metabolism, such as hepcidin and erythroferrone, have been proposed as potential indicators of blood doping [10, 11, 12, 13]. Additionally, transcriptomic biomarkers have shown promise in detecting rHuEPO use [14, 15, 16]. For example, the expression of two genes associated with reticulocyte production, *ALAS2* and *CA1*, has been demonstrated to respond robustly to rHuEPO administration, offering new avenues for detection.

Moreover, metabolomics (which is the comprehensive analysis of small molecules in biological samples) has emerged as a potential tool for identifying novel biomarkers of blood doping [17]. With untargeted metabolomics, Lima et al. discovered several metabolites altered by rHuEPO administration [18]. However, the untargeted approach presents some issues, notably in metabolite identification, analytical sensitivity, and reproducibility, which can complexify the translation of findings into practical applications and/or official guidelines. In contrast, targeted (or multitargeted) metabolomics, which focuses on monitoring a predefined set of metabolites with high sensitivity and specificity, offers a more accurate analytical alternative. Despite its advantages, this approach has not been extensively applied to the investigation of candidate biomarkers for blood doping, particularly in the context of rHuEPO. Particularly noteworthy are polar metabolites—such as amino acids, purine derivatives, nucleotides, and carnitines—which play key roles in cellular energy metabolism, redox balance, and nucleotide turnover. Increasing evidence suggests that some of these metabolites not only reflect physiological states but also act as signaling molecules or exhibit hormone‐like functions. To support their investigation, numerous generic analytical methods, primarily based on hydrophilic interaction liquid chromatography coupled with tandem mass spectrometry (HILIC‐MS/MS), have been developed for the sensitive and simultaneous quantification of a broad range of polar metabolites [19, 20, 21]. These methods enable comprehensive coverage of key metabolic pathways including glycolysis, the TCA cycle, amino acid metabolism, nucleotide turnover, and one‐carbon metabolism.

In this study, a targeted metabolomic method focusing on polar endogenous metabolites was implemented to characterize candidate biomarkers of rHuEPO administration. Samples from two independent administration studies were analyzed, revealing promising biomarkers that could complement existing ABP markers and improve the detection of stimulated erythropoiesis.

## 2 Material and Methods

### 2.1 Chemical and Reagents

Acetonitrile (ACN), methanol (MeOH), and formic acid (FA, 99%), all of UHPLC–MS grade, were obtained from Biosolve BV (Valkenswaard, Netherlands). Ammonium formate (99%) was purchased from Sigma‐Aldrich (St. Louis, USA). Ultrapure water was produced using a Milli‐Q system from Millipore (Burlington, USA). The Mass Spectrometry Metabolite Library of Standards—MSMLS was obtained from Sigma‐Aldrich.

### 2.2 Sample Preparation

The preparation of plasma and serum samples involved protein precipitation via monophasic extraction of polar metabolites as described in [21]. Aliquots of 25‐μL plasma were transferred to 1.5‐mL Eppendorf tubes. Subsequently, 150 μL of cold MeOH:ACN (50:50) (stored at −20°C) was added to each tube. The mixture was briefly vortexed and then incubated at −20°C for 1 h. Following incubation, the samples were centrifuged at 10,000 rpm and 4°C for 15 min. Finally, 100 μL of the supernatant was collected, transferred into LC–MS vials with inserts, and injected into the UHPLC–MS system.

### 2.3 Targeted Metabolome Analysis

Plasma and serum extracts were analyzed using hydrophilic interaction chromatography coupled with electrospray ionization tandem mass spectrometry (HILIC‐ESI‐MS/MS) in positive ionization mode. The analyses were conducted on an Acquity UPLC I‐Class system coupled to a Xevo‐TQ S triple quadrupole mass spectrometer (Waters, Milford, MA, USA). Chromatographic separation was achieved using an Acquity BEH Amide column (100 mm × 2.1 mm, 1.7 μm) with a BEH Amide VanGuard precolumn (2.1 × 5 mm, 1.7 μm) (Waters) as described in [19, 20, 22]. The mobile phase consisted of 20‐mM ammonium formate with 0.1% formic acid in water (Phase A) and 0.1% formic acid in acetonitrile (Phase B). A linear gradient was applied, starting at 95% B (0–1.5 min) and decreasing to 45% B (1.5–17 min), followed by a 2‐min hold. The initial conditions were restored during a 5‐min post‐run for column re‐equilibration. The flow rate was set at 400 μL/min, column temperature at 25°C, and injection volume at 2 μL. Electrospray ionization source parameters were optimized as follows: source temperature, 150°C; desolvation temperature, 600°C; desolvation gas flow, 800 L/h; cone gas flow, 150 L/h; cone voltage, 15 V; and capillary voltage, 2 kV. Data were acquired in multiple reaction monitoring (MRM) mode. MRM transitions were optimized through direct analysis of pure chemical standards, yielding two transitions per metabolite whenever possible [20]. Retention times for each metabolite were determined by direct injection of the standards. The MRM transitions for each metabolite and their corresponding retention times are reported in Table S1. In total, 196 transitions were monitored. Dwell times were automatically calculated by the instrument software to ensure a minimum of 10 data points across each chromatographic peak, balancing sensitivity, and temporal resolution. Scheduled MRM was employed, allowing the instrument to allocate longer dwell times within defined retention time windows, thus improving the overall data quality for complex biological matrices. The list of metabolites includes key intermediates in amino acid metabolism, carbohydrate processing, nucleotide synthesis, lipid metabolism, and energy production.

### 2.4 Administration Study Samples

Samples from two independent administration studies involving healthy Caucasian male participants were used for targeted metabolomic analyses. In both studies, participants were asked to abstain from any dangerous activities, maintain a regular sleep schedule without late‐night outings, completely cease consumption of cigarettes, medications, or drugs, and strongly limit intake of coffee, tea, alcohol, and colas. Details of the studies, including ethical approvals, have been described in previous publications [23, 24].

#### 2.4.1 Continuous Erythropoietin Receptor Activator (CERA) Administration Study

Six participants, aged 20–28 years (mean ± SD: 23.0 ± 2.97 years) with a mean body mass index (BMI) of 23.3 ± 1.48 kg/m^2^, received a single injection of 200‐μg CERA (MIRCERA, Roche Pharma AG, Reinach, Switzerland). Blood samples were systematically collected over 4‐day postinjection. Additional samples were collected on Days 6, 8, 10, 13, 16, 20, 24, and, for four participants, on Day 27. Two samples per subject were collected: one in a serum gel tube (7.5 mL) and the other in an EDTA‐coated tube (2.6 mL) (Sarstedt, Nümbrecht, Germany). A complete red blood cell count and reticulocyte quantification (XT‐2000i analyzer, Sysmex, Norderstedt, Germany) were performed on whole EDTA blood samples to monitor hematological changes following CERA administration. Following full blood count, EDTA samples as well as serum tubes were centrifuged at 1500 ×*g* for 15 min. Serum and plasma samples were then aliquoted and stored at −20°C.

#### 2.4.2 Epoetin Delta Administration Study

Six participants, aged 22–33 years (mean ± SD: 27.0 ± 4.1 years) with a mean BMI of 23.9 ± 2.66 kg/m^2^, received intravenous (*n* = 3) or subcutaneous (*n* = 3) injections of epoetin delta (Dynepo, Dynepo Shire Pharmaceuticals, Basingstoke, UK). Each subject was administered 5000 IU on Days 1, 3, and 5, followed by 2500 IU on Days 7, 9, and 11. Blood samples (EDTA and serum tubes) were collected before the first injection, over five consecutive days following the first injection, and on Days 7, 9, 11, 14, and 17.

### 2.5 Data Processing and Data Quality

Raw LC–MS/MS metabolomic data were processed using TargetLynx software (version 4.2, Waters). Peak integration and peak area extraction for each polar metabolite were performed based on their extracted ion chromatograms (EICs) corresponding to the monitored MRM transitions. Data quality was assessed using pooled quality control (QC) samples analyzed periodically (every five or eight samples) throughout the entire randomized batch. The obtained tables (containing peak areas of detected metabolites across all samples) were exported to the Excel‐based Metabodrift normalization tool (version 1.1) for signal intensity drift correction using the LOESS algorithm [25]. Peaks with CV > 20% across QC samples were removed from further analysis.

### 2.6 Statistical Analysis

The generalized least squares (GLS) model from R package ‘nmle’ [26] was used to investigate the fluctuation of the various polar metabolites over time for both administration studies. The use of GLS (*gls (metabolite~day*,*corr = corCAR1*(*value = 0.5, form = ~day|id*)) allowed for the correlation between repeated measurements over time, providing a precise and accurate model of metabolomic data. The Benjamini–Hochberg method corrected the obtained *p*‐values for the false discovery rate (adjusted *p* < 0.05). The ‘*predict*’ function was then used to compute the predictive mean of significantly altered polar species over time.

Correlations were calculated using Pearson's coefficient test method. A *p‐*value < 0.05 was considered statistically significant. Statistical analyses and descriptive comparisons were performed with R Studio software.

## 3 Results

### 3.1 CERA Study

Following the analysis of plasma samples collected during the CERA administration study, a total of 129 polar metabolites were kept for statistical analysis after applying signal drift correction and QC filtering using pooled QC samples. The application of GLS models allowed for the identification of metabolites showing significant temporal variation in response to CERA administration.

Five metabolites exhibited statistically significant changes at least at one point (Table 1). These included inosine, guanosine, hypoxanthine, arachidonyl‐L‐carnitine, and oleoyl‐L‐carnitine. Notably, inosine and hypoxanthine showed the most pronounced and consistent alterations, with significant increases observed as early as Day 4 postadministration. Hypoxanthine remained significantly elevated up to Day 13, suggesting a sustained metabolic response. Arachidonyl‐L‐carnitine and oleoyl‐L‐carnitine were increased at Day 13, indicating potential involvement in later stages of erythropoietic stimulation or recovery.

**TABLE 1:** List of metabolites identified as significantly altered following single CERA administration. Day represents the time points with significant alteration, and the fold‐change represents the magnitude of change versus Day 0 (control time point).

Metabolite	Day	*p*‐value	Fold‐change vs. 0
Inosine	4	< 0.001	20.4
Guanosine	4	< 0.001	8.7
Hypoxanthine	4, 8, and 13	< 0.001, < 0.001, and < 0.05	5.4, 5.4, and 4.4
Arachidonyl‐L‐carnitine	13	< 0.05	3.4
Oleoyl‐L‐carnitine	13	< 0.05	2.4

Although guanosine was initially identified as significantly altered at Day 4, a careful inspection of the chromatograms revealed a low and unreliable signal for this metabolite. This limited detectability is likely due to its low endogenous abundance. Consequently, guanosine was not considered a robust candidate for monitoring CERA‐induced effects and was excluded from further interpretation.

**FIGURE 1:**
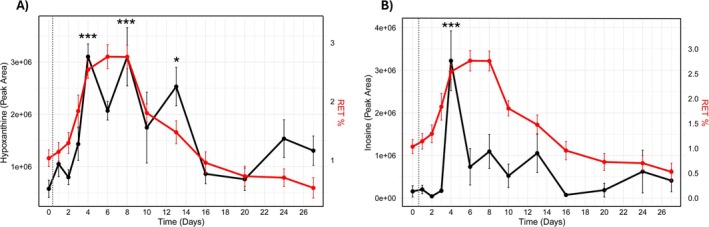
Fluctuations of hypoxanthine (A) and inosine (B) in relation to reticulocytes percentage in red. Dashed line indicates CERA administration. *** indicates *p* < 0.001 and * indicates *p* < 0.05.

Among all identified features, inosine and hypoxanthine stood out as the most responsive metabolites to CERA administration, exhibiting the highest fold changes over time. Their longitudinal profiles are presented in Figure 1, while the remaining significant metabolites are provided in Figure S1.

Given that the percentage of reticulocytes (RET%) is both a diagnostic marker of erythropoiesis stimulation and a key parameter in the hematological module of the ABP, the trajectories of inosine and hypoxanthine were compared to that of RET%. Inosine displayed a sharp increase, peaking at Day 4 post‐CERA administration, and remained elevated through Day 13, indicating a sustained metabolic response. Hypoxanthine followed a similar pattern, with a high value at Day 4 and persistently elevated levels until Day 13, although the increase at Days 6 and 10 did not reach statistical significance. Interestingly, the RET% response mirrored these dynamics, peaking between Days 4 and 8, which aligns with the window of maximal erythropoietic activity following CERA exposure. However, the maximal increase in RET% was less pronounced (2.7‐fold) compared to that observed for both metabolites. This temporal alignment was supported by moderate correlations between hypoxanthine and RET% (*r* = 0.52, *p* < 0.001), and between inosine and RET% (*r* = 0.35, *p* = 0.002). Furthermore, inosine and hypoxanthine levels were strongly correlated with each other (*r* = 0.61, *p* < 0.001), reinforcing the idea of a shared metabolic signature associated with erythropoietic stimulation.

### 3.2 Dynepo Study

In a second study involving repeated administration of Dynepo (with injections on Days 1, 3, 5, 7, 9, and 11), 119 polar metabolites passed QC check criteria and were included in the final dataset. Consistent with findings from the CERA study, inosine and hypoxanthine displayed the most substantial responses to erythropoietic stimulation. Inosine levels were the highest on Day 7, showing a tenfold increase compared to baseline (*p* < 0.001), while hypoxanthine exhibited significant increases on Days 5 and 7, with fold changes of 3.7 and 3.3, respectively (*p* < 0.001 for both; Table 2).

**TABLE 2:** List of metabolites identified as significantly altered following multiple Dynepo administrations. Day represents the time points with significant alteration, and the fold‐change represents the magnitude of change versus Day 0 (control time point).

Metabolite	Day	*p*‐value	Fold‐change vs. 0
Inosine	7	< 0.001	10
Hypoxanthine	5 and 7	< 0.001 and < 0.001	3.7 and 3.3
Nicotinamide	5	< 0.001	1.6
Arachidonyl‐L‐carnitine	9 and 11	< 0.001 and < 0.05	1.7 and 1.5
Oleoyl‐L‐carnitine	9 and 11	< 0.05 and < 0.05	1.4 and 1.4
Phenylalanine	9, 11, 14, and 17	< 0.05, < 0.001, < 0.001, and < 0.001	1.3, 1.4, 1.3, and 1.4
Leucine	11, 14, and 17	< 0.001, < 0.001, and < 0.05	1.3, 1.2, and 1.2

**FIGURE 2:**
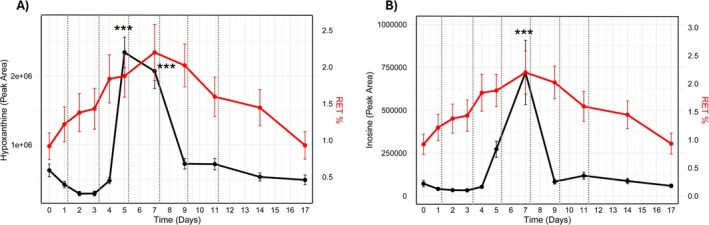
Fluctuations of hypoxanthine (A) and inosine (B) in relation to reticulocytes percentage in red. Dashed lines indicate Dynepo administration. *** indicates *p* < 0.001 and * indicates *p* < 0.05.

Once again, when plotted against the percentage of reticulocytes (RET%), the longitudinal patterns of both inosine and hypoxanthine followed a comparable temporal trajectory to RET%, supporting their potential utility as indirect markers of erythropoietic stimulation (Figure 2). RET% increased progressively throughout the treatment phase, with a peak at Day 7, overlapping with the metabolite response. Similar to the CERA study, the maximal increase was less pronounced than that observed for inosine and hypoxanthine (2.4‐fold). In line with this observation, the correlation between inosine and hypoxanthine was high (*r* = 0.78, *p* < 0.001), while moderate correlations were observed between inosine and RET% (*r* = 0.30, *p* = 0.016), and between hypoxanthine and RET% (*r* = 0.25, *p* = 0.048).

Several additional metabolites showed statistically significant, but more moderate, changes. Nicotinamide levels increased modestly at Day 5 (fold‐change 1.6, *p* < 0.001). Arachidonyl‐L‐carnitine and oleoyl‐L‐carnitine were slightly higher at Days 9 and 11, with fold changes ranging from 1.4 to 1.7. Moreover, branched‐chain and aromatic amino acids such as phenylalanine and leucine also increased from Day 9 onwards, likely reflecting broader metabolic adaptations to enhanced erythropoiesis (Table 2). The longitudinal changes of these metabolites are presented in Figure S2.

### 3.3 Comparison of Plasma and Serum for Hypoxanthine and Inosine Measurement

**FIGURE 3:**
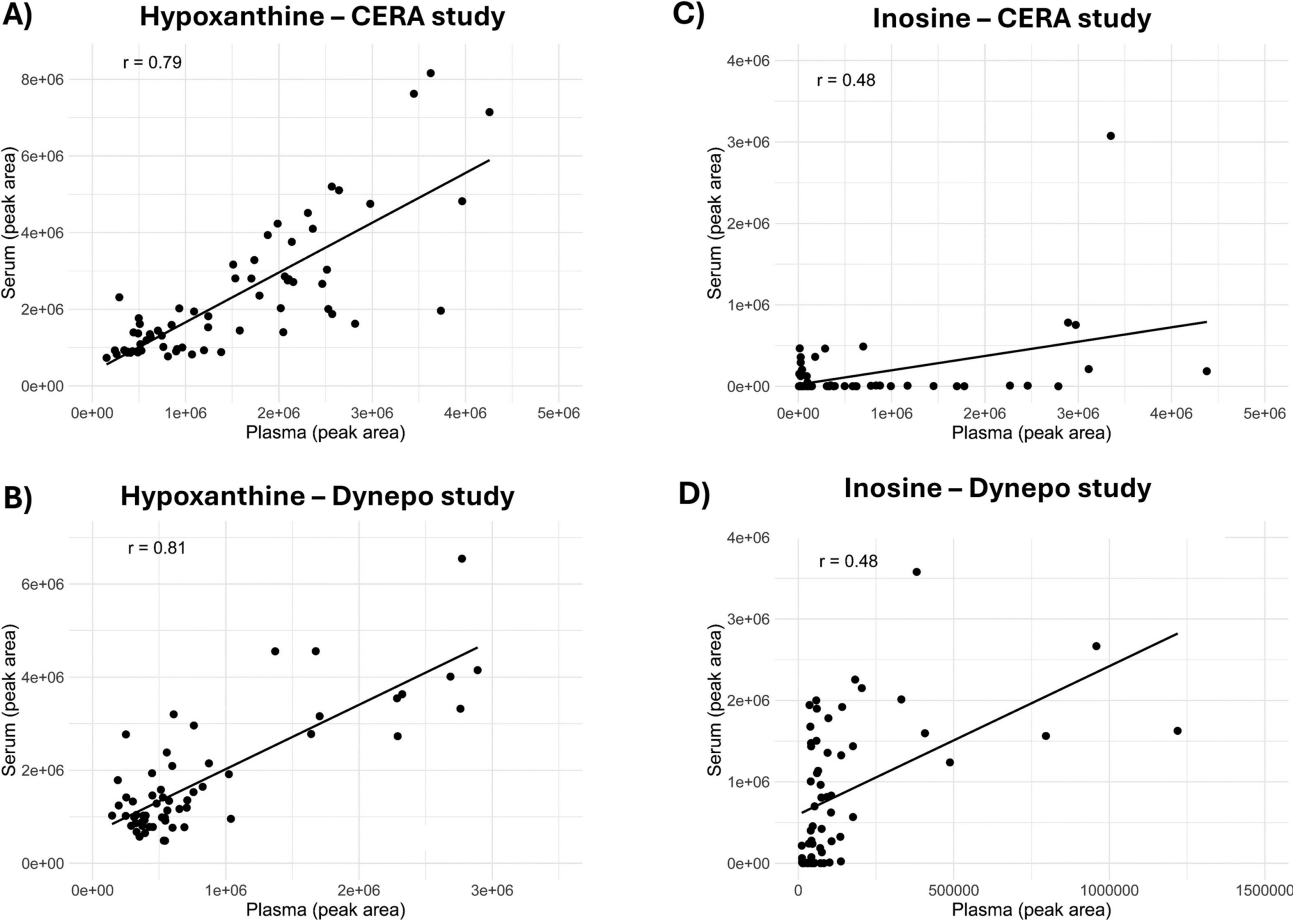
Correlation of peak area of hypoxanthine (A and B) and inosine (C and D) between plasma and serum for CERA and Dynepo administration studies.

Since serum is currently gaining importance as the preferred matrix for biomarkers monitoring in the context of the ABP, corresponding serum samples from both CERA and Dynepo administration studies were also analyzed with the targeted metabolomic method. This approach aimed to determine whether similar metabolic responses could also be observed in serum and to assess the level of agreement between serum and plasma samples for both biomarkers, namely, inosine and hypoxanthine. Hypoxanthine showed good correlation between the two matrices in both studies, with significant correlations (i.e., *r* = 0.79 (*p* < 0.001) for CERA and *r* = 0.81 (*p* < 0.001) for Dynepo). The longitudinal profiles for hypoxanthine levels were therefore comparable in serum and plasma; however, the signal intensity in serum appeared approximately twofold higher (Figure 3 and S3). In contrast, inosine exhibited no correlation between serum and plasma, with a coefficient lower than 0.5 (i.e., *r* = 0.48), suggesting that coagulation processes may affect inosine levels in serum and limit its reliability as a marker in this matrix.

## 4 Discussion

This study demonstrates the applicability and sensitivity of targeted metabolomics for detecting metabolic changes following rHuEPO administration, using both CERA and Dynepo as model compounds. A key advantage of targeted over untargeted metabolomics lies in its ability to provide greater confidence in peak monitoring, thanks to the use of authentic standards and optimized acquisition parameters [27]. This approach ensures precise and sensitive detection of selected metabolites, enabling robust longitudinal profiling and the reliable detection of biologically meaningful changes in response to interventions such as EPO stimulation.

Among the most striking findings, inosine and hypoxanthine emerged as the most responsive metabolites following administration of both CERA and Dynepo. These purine degradation products showed concordant and substantial increases across both studies, with fold changes exceeding tenfold for inosine and threefold to fivefold for hypoxanthine. The consistency of these responses across independent datasets underscores the reliability of these metabolic markers and strengthens their potential relevance as indicators of erythropoietic activity.

**FIGURE 4:**
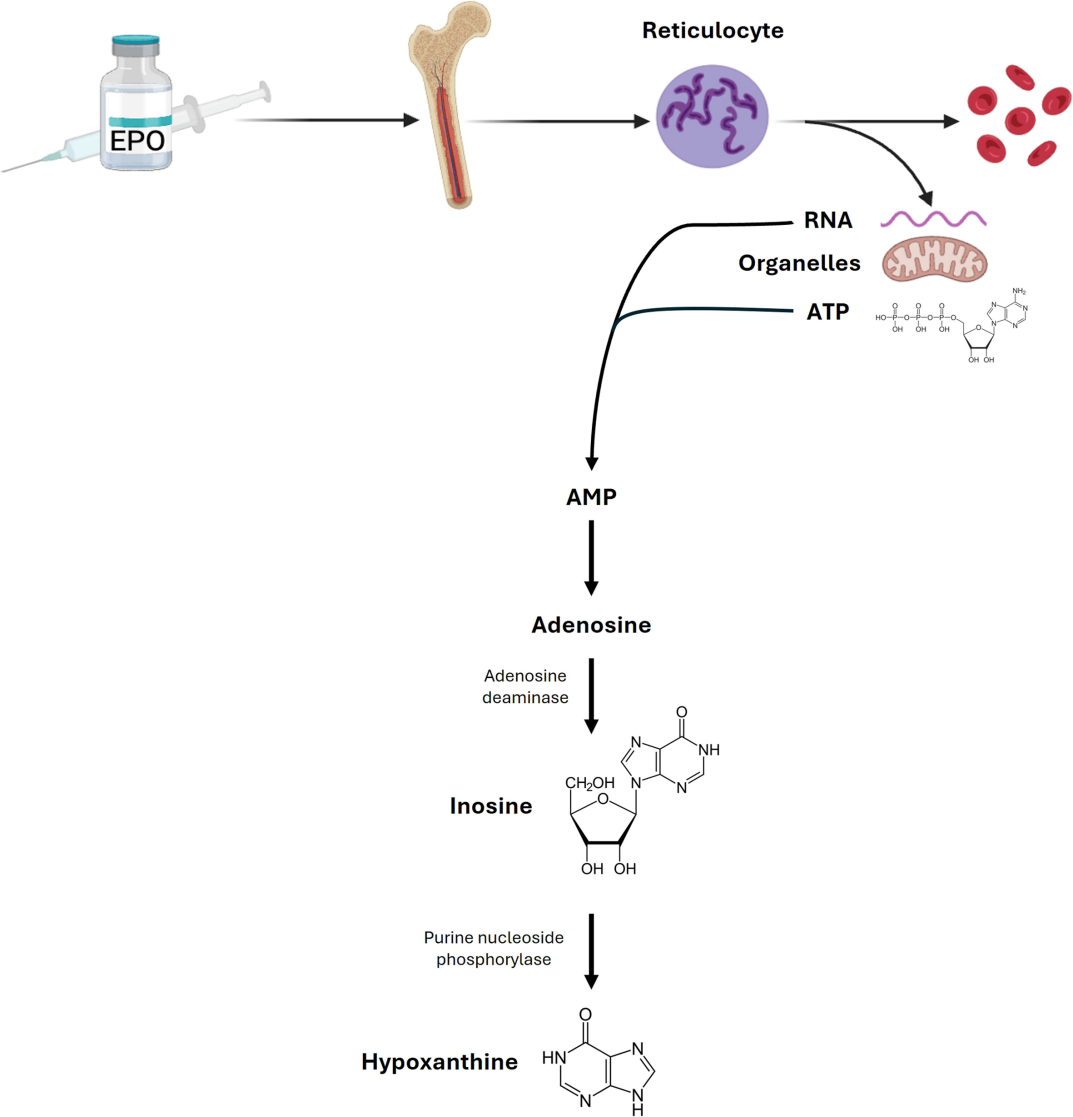
Relationship between reticulocyte production and maturation with purine metabolism and production of inosine and hypoxanthine.

The temporal patterns of inosine and hypoxanthine were notably aligned with fluctuations in RET%, a well‐established hematological marker of erythropoiesis and a core component of the ABP. In both studies, peak increases in these metabolites coincided with or slightly preceded the rise in RET%, suggesting that they may reflect upstream metabolic shifts associated with enhanced erythropoietic drive. Correlations between hypoxanthine and RET% (*r* = 0.52 in CERA; *r* = 0.25 in Dynepo), and between inosine and RET% (*r* = 0.35 in CERA; *r* = 0.30 in Dynepo), support a moderate yet consistent biological association. Interestingly, the two most responsive metabolites identified across both studies, inosine and hypoxanthine, belong to the purine family and are sequentially linked within the same metabolic pathway. Their coordinated increase following EPO administration suggests a shared biological origin. We hypothesize that this response is closely related to reticulocyte dynamics (Figure 4). Reticulocytes, as immature red blood cells, retain residual RNA and remain metabolically active [28]. During their maturation into erythrocytes, they undergo extensive remodeling, including the degradation of RNA and other organelles that are no longer required [29]. This RNA breakdown releases purine nucleotides, which are subsequently catabolized into inosine and hypoxanthine. Additionally, the energy demand associated with reticulocyte maturation, driven by ATP consumption, may further contribute to elevated purine turnover [30]. Supporting this hypothesis, we observed that the temporal profiles of inosine and hypoxanthine mirrored that of the RET%, with peak levels aligning during the window of maximal erythropoietic activity, reinforcing the idea that these metabolic changes are closely linked to erythropoiesis. Notably, the magnitude of the response for both metabolites appeared to be stronger than that of RET%, suggesting that inosine and hypoxanthine may serve as highly sensitive indicators of erythropoietic stimulation. While RET% remains a key marker in the ABP for detecting blood manipulations [31, 32], monitoring inosine and hypoxanthine fluctuations in parallel could enhance its sensitivity. The combined measurement of RET% with inosine and hypoxanthine levels might improve the detection of subtle or early erythropoietic responses that could otherwise remain below the threshold of detection when relying solely on reticulocyte counts. Interestingly, xanthine, a downstream metabolite in the purine degradation pathway, did not exhibit a significant increase following EPO administration (data not shown). This may reflect a transient accumulation of upstream intermediates (inosine and hypoxanthine) due to localized production and rapid turnover in maturing reticulocytes, or a rate‐limiting step at the level of xanthine oxidoreductase, which catalyzes the conversion of hypoxanthine to xanthine. These observations highlight the importance of examining early purine intermediates as more responsive markers of acute erythropoietic activity.

In addition to purine metabolism, carnitine derivatives such as arachidonyl‐L‐carnitine and oleoyl‐L‐carnitine also exhibited significant increases, particularly in the later phases of both studies. These metabolites may reflect broader metabolic adaptations linked to lipid mobilization, mitochondrial function, or erythrocyte turnover [33, 34]. Furthermore, more subtle changes were observed in nicotinamide, phenylalanine, and leucine, suggesting systemic metabolic reprogramming under erythropoietic stimulation, potentially involving amino acid metabolism and NAD+ biosynthesis [35]. Nevertheless, it is important to note that plasma metabolite levels represent a systemic snapshot influenced by multiple tissues and cellular compartments. The observed changes in carnitine derivatives and amino acids likely reflect integrated metabolic responses involving transport processes between cells and tissues rather than direct measures of intracellular concentrations. Therefore, while these metabolites suggest broader metabolic adaptations linked to erythropoietic stimulation, further targeted studies are needed to elucidate the specific cellular and tissue contributions and transport mechanisms underlying these plasma changes.

An important methodological consideration was the comparison of plasma and serum as biological matrices for metabolite monitoring. While serum is the currently preferred matrix for biomarker monitoring for endocrine and steroidal modules in the ABP, our findings suggest that plasma may offer certain advantages, particularly for inosine measurement. Hypoxanthine showed strong agreement between matrices in both studies, with correlation coefficients exceeding 0.79. However, inosine displayed poor concordance (*r* = 0.48). It suggests that coagulation, which occurs in the serum samples, influences the level of inosine. Indeed, inosine is less stable than hypoxanthine and is prone to enzymatic breakdown (e.g., by purine nucleoside phosphorylase) during the clotting process that occurs in serum preparation. This enzymatic activity may significantly alter inosine levels in serum, masking its response to EPO administration. In contrast, hypoxanthine, being further downstream in the purine catabolic pathway, is less susceptible to enzymatic variability. In this direction, plasma matrix has been suggested as the matrix of choice for metabolomic studies to avoid alteration of serum metabolite levels due to coagulation [36, 37]. Moreover, the gels used in serum separator tubes may introduce biases in the metabolome and potentially influence inosine levels through adsorption or delayed processing effects [38].

These candidate biomarkers offer several advantages, including their substantial amplitude of response and the ability to detect changes using as little as 25 μL of plasma. The use of EDTA plasma, already routinely collected for the ABP, ensures full compatibility with existing anti‐doping workflows and maximizes the utility of archived samples. Together, these findings highlight the power of targeted metabolomics to reveal reproducible metabolic signatures of EPO administration. The strong and consistent responses of inosine and hypoxanthine, their temporal alignment with erythropoietic markers, and their detection across two independent studies support their potential as candidate biomarkers for erythropoiesis monitoring.

However, critical validation steps remain necessary before these candidate biomarkers can be implemented in routine anti‐doping applications. These include the development and validation of a robust quantitative assay for plasma to reproduce these findings, as well as thorough assessments of intra‐ and inter‐individual variability under baseline conditions. Understanding the natural fluctuation of inosine and hypoxanthine within individuals over time will be critical for establishing baseline levels and refining their application in longitudinal monitoring approaches.

Importantly, the specificity of inosine and hypoxanthine as biomarkers of exogenous EPO stimulation must be evaluated in the context of potential confounding factors. In this study, volunteers did not engage in exercise, so observed metabolite changes are attributable to EPO administration. However, altitude exposure is known to stimulate erythropoiesis and can lead to transient increases in RET%, potentially mimicking the effects of EPO. Similarly, intense exercise induces ATP breakdown and purine metabolism, which could also elevate circulating levels of inosine and hypoxanthine independent of EPO use [39, 40, 41]. It is well recognized in the anti‐doping community that intense in‐competition exercise causes acute plasma volume shifts, leading to transient fluctuations in concentration‐based biomarkers. This physiological effect is why blood samples are generally not collected within 2‐h postexercise to avoid confounding influences. Consequently, the impact of exercise‐induced changes in plasma volume and purine metabolism must be carefully considered and validated before using inosine and hypoxanthine as doping biomarkers. Additionally, some dietary supplements may contain inosine or purine precursors that could influence plasma concentrations. Comprehensive studies accounting for these physiological and environmental variables will be essential to establish the specificity, robustness, and interpretability of these markers in anti‐doping contexts.

The collection of venous blood samples (whether in EDTA or serum tubes) within the anti‐doping context poses several logistical challenges, including the requirement for trained phlebotomists and the substantial costs associated with cold‐chain shipping to anti‐doping laboratories. As an alternative, the use of dried blood spots (DBS) offers a minimally invasive, cost‐effective, and logistically simpler solution. DBS sampling does not require specialized personnel for collection, enables easy storage and transport without the need for refrigeration. Notably, analytes like hypoxanthine exhibit excellent stability in dried matrices, largely due to the absence of water, which limits enzymatic activity and degradation [42]. Taken together, this approach may represent a practical and reliable alternative for the longitudinal monitoring of inosine and hypoxanthine within the ABP framework.

## 5 Conclusion

This study demonstrates the value of targeted metabolomics for detecting metabolic responses to rHuEPO administration, using both CERA and Dynepo as model compounds. Inosine and hypoxanthine emerged as consistently responsive purine metabolites, showing substantial and coordinated increases across two independent studies. Their temporal alignment with RET%, a core marker of erythropoiesis, suggests a close link to reticulocyte maturation and purine turnover, supporting their potential utility as sensitive indicators of erythropoietic stimulation.

Importantly, plasma proved to be a more suitable matrix than serum for inosine monitoring due to its greater metabolic stability, reinforcing the relevance of EDTA plasma samples already used in anti‐doping workflows.

While these findings are promising, further validation is required, including quantitative assay development, evaluation of biological variability, and assessment of potential confounding factors such as altitude, exercise, and supplementation. Ultimately, integrating such metabolic markers into ABP frameworks holds the potential to significantly enhance its sensitivity, specificity, and interpretative power. This would provide a valuable complement to the existing hematological parameters, offering a more comprehensive and physiologically informed approach to detecting blood manipulation.

## Conflicts of Interest

The authors declare no conflicts of interest.

## Supporting information


**Figure S1:** Peak area of (A) guanosine, (B) oleoyl‐L‐carnitine, and (C) arachidonyl‐L‐carnitine in plasma following CERA administration at Day 0 (indicated as dashed line).
**Figure S2:** Peak area of (A) nicotinamide, (B) oleoyl‐L‐carnitine, (C) arachidonyl‐L‐carnitine, (D) phenylalanine, and (E) leucine in plasma following Dynepo administration at Day 1, 3, 5, 7, 9, and 11 (indicated as dashed line).
**Figure S3:** Peak area of (A) and (B) hypoxanthine and (C) and (D) inosine in serum after CERA and Dynepo administration.
**Table S1:** MRM list for the metabolites included into the method.

## Data Availability

The data that support the findings of this study are available from the corresponding author upon reasonable request.
