# Supplementary material for: Identification of Candidate Blood Biomarkers of Recombinant Human Erythropoietin Administration Using Targeted Polar Metabolomics by HILIC‐MS/MS
Source: Drug Test Anal. 2025 Aug 31;17(12):2323–32. doi: 10.1002/dta.3943 (PMC12689250; doi:10.1002/dta.3943)
Supplement: Supplementary file 1 — Figure S1: Peak area of (A) guanosine, (B) oleoyl‐L‐carnitine, and (C) arachidonyl‐L‐carnitine in plasma following CERA administration at Day 0 (indicated as dashed line). Figure S2: Peak area of (A) nicotinamide, (B) oleoyl‐L‐carnitine, (C) arachidonyl‐L‐carnitine, (D) phenylalanine, and (E) leucine in plasma following Dynepo administration at Day 1, 3, 5, 7, 9, and 11 (indicated as dashed line). Figure S3: Peak area of (A) and (B) hypoxanthine and (C) and (D) inosine in serum after CERA and Dynepo administration. Table S1: MRM list for the metabolites included into the method. [file DTA-17-2323-s001.docx]

**Supplementary data**

**Discovery of candidate blood biomarkers of recombinant human erythropoietin administration using targeted metabolomics**

**Table of contents**

**Figure S1** – Peak area of A) guanosine, B) oleoyl-L-carnitine and C) arachidonyl-L-carnitine in plasma following CERA administration at day 0 (indicated as dashed line).

**Figure S2 –** Peak area of A) nicotinamide, B) oleoyl-L-carnitine, C) arachidonyl-L-carnitine, D) phenylalanine and E) leucine in plasma following Dynepo administration at day 1, 3, 5, 7, 9 and 11 (indicated as dashed line).

**Figure S3** – Peak area of A) and B) hypoxanthine and C) and D) inosine in serum after CERA and Dynepo administration.

**Table S1** – MRM list for the metabolites included into the method


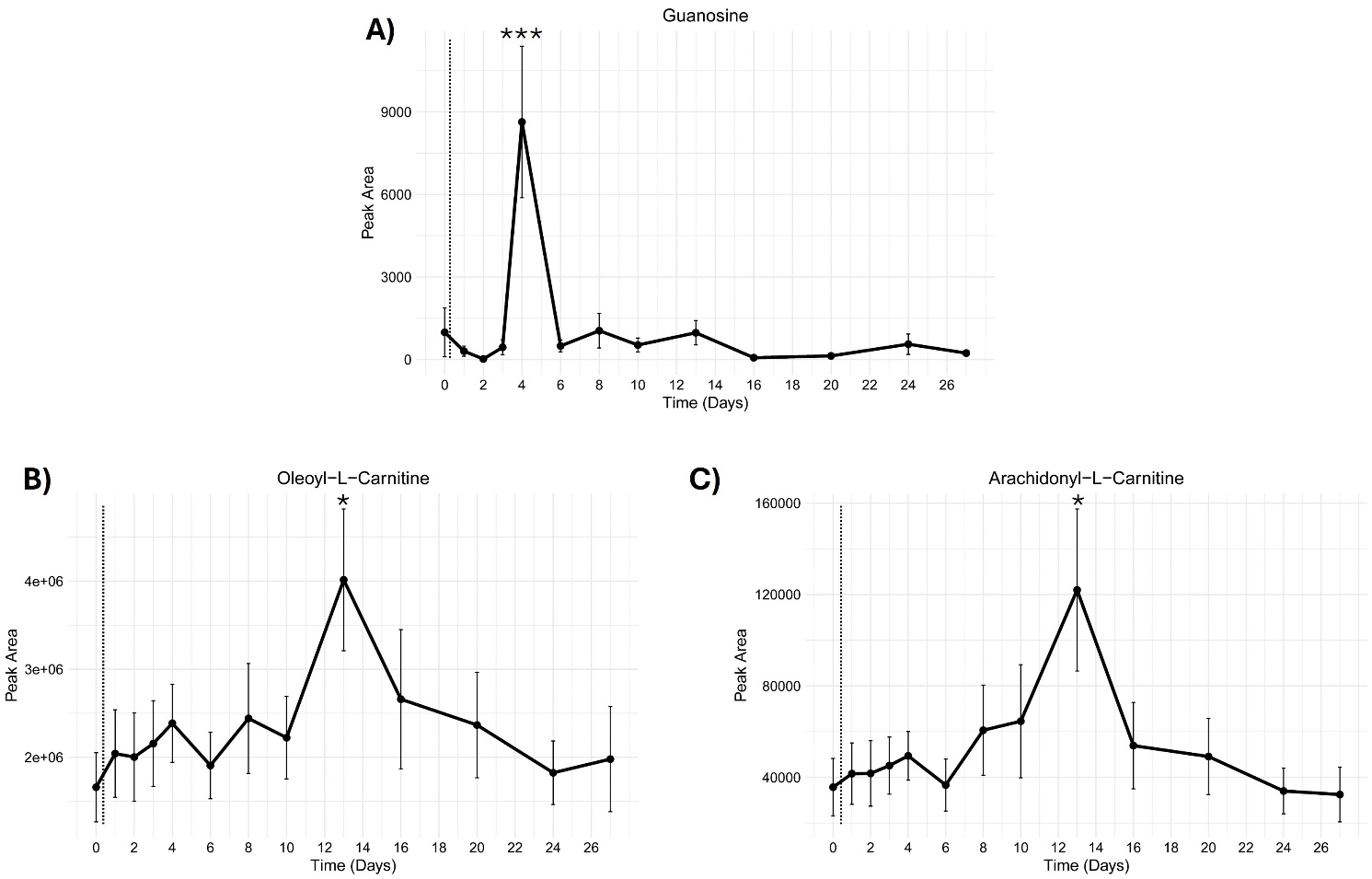


**Figure S1**

**
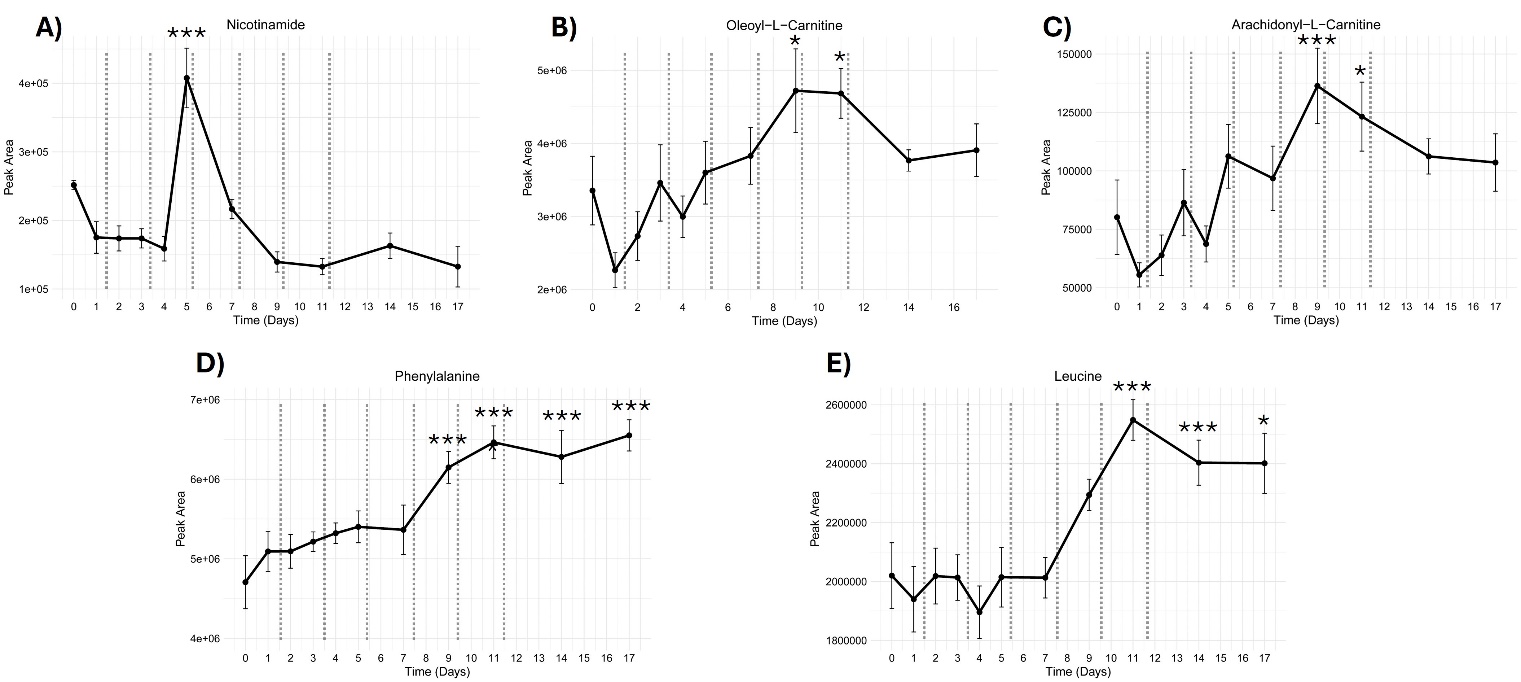
**

**Figure S2**

**
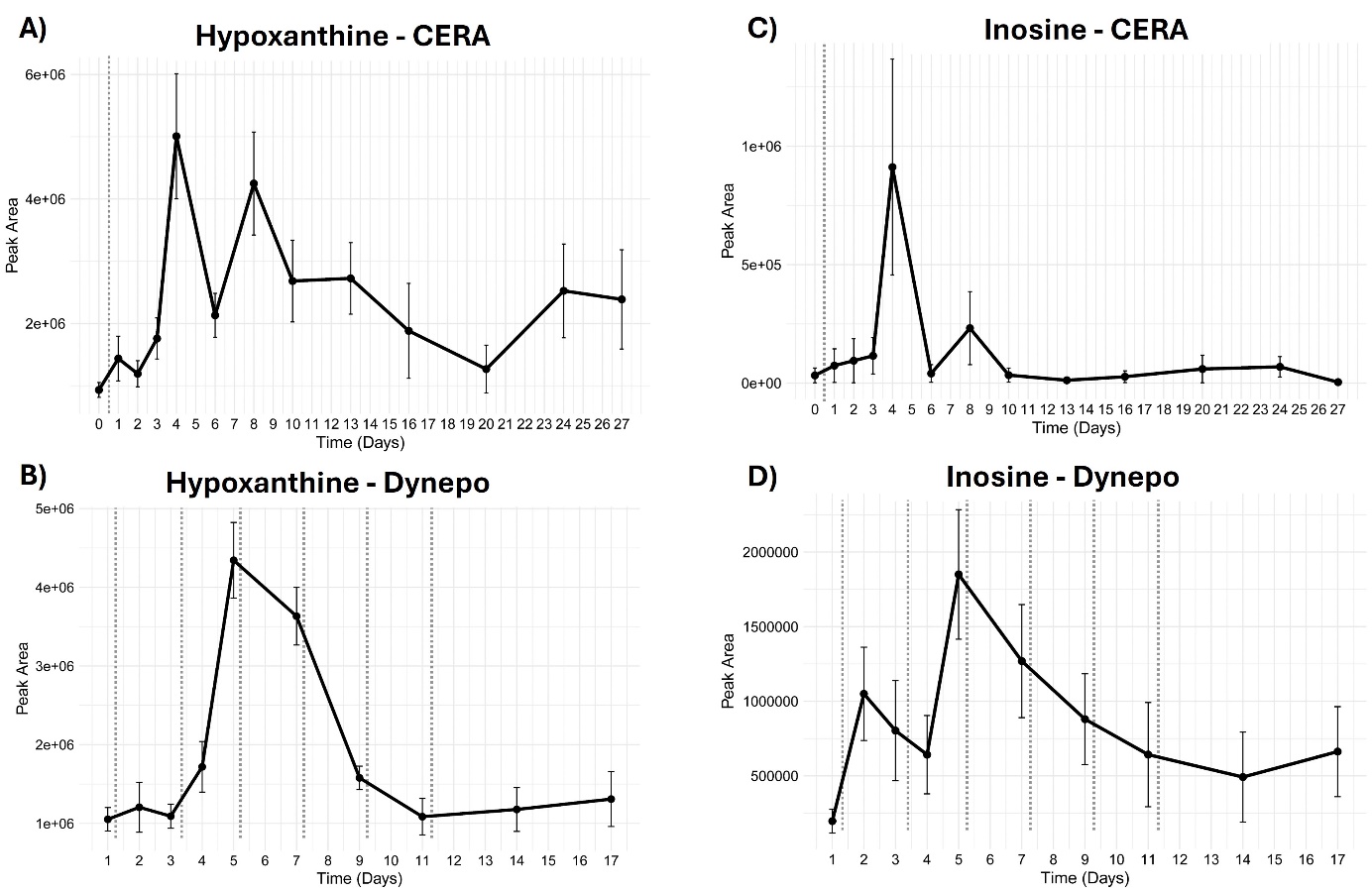
**

**Figure S3**

**Table S1** – MRM list for the metabolites included into the method

| **Compound** | **Precursor ion (m/Z)** | **Product ion (m/z)** | **Collision energy (eV)** | **Retention time (min)** |
| --- | --- | --- | --- | --- |
| 1-Aminocyclopropanecarboxylate | 102.04 | 56.00 | 12 | 8.15 |
| 1-Methyl-L-histidine | 170.08 | 123.99 ; 83.17 | 15 ; 20 | 10.20 |
| 1-Methyladenosine | 282.11 | 150.06 ; 57.09 | 20 ; 30 | 3.04 |
| 1-Methylnicotinamide | 138.07 | 79.11 ; 95.25 | 20 ; 20 | 5.47 |
| 2-Aminoisobutyrate | 104.06 | 58.24 ; 41.39 | 15 ; 20 | 7.59 |
| 2-Oxobutanoate | 103.03 | 61.84 ; 77.04 | 15 ; 15 | 4.08 |
| 2-Phosphoglycerate | 186.99 | 101.94 | 15 | 10.90 |
| 3-Methoxytyramine | 168.09 | 151.16 ; 91.14 | 15 ; 20 | 4.46 |
| 3-Methylhistamine | 126.09 | 109.11 ; 96.16 | 15 ; 20 | 9.33 |
| 3-Sulfinoalanine | 154.00 | 74.28 ; 44.10 | 15 ; 15 | 9.95 |
| 3,5-Diiodo-L-thyronine | 525.89 | 479.62 ; 352.96 | 15 ; 30 | 5.33 |
| 4-Acetamidobutanoate | 146.07 | 86.09 ; 42.97 | 15 ; 20 | 1.52 |
| 4-Guanidinobutanoate | 146.08 | 86.15 ; 60.12 | 15 ; 15 | 6.02 |
| 4-Imidazoleacetate | 127.04 | 81.07 ; 54.06 | 15 ; 20 | 7.65 |
| 4-Pyridoxate | 184.05 | 166.13 ; 148.11 | 15 ; 20 | 2.27 |
| 5-Aminolevulinate | 132.05 | 86.15 ; 114.20 | 15 ; 15 | 7.03 |
| 5-Hydroxyindoleacetate | 192.05 | 145.87 ; 91.19 | 15 ; 30 | 1.15 |
| 5-Hydroxylysine | 163.10 | 82.12 ; 128.27 | 15 ; 15 | 11.55 |
| 5-Methylcytosine | 126.05 | 109.05 ; 83.08 | 15 ; 15 | 4.84 |
| 6-Hydroxynicotinate | 140.02 | 122.03 ; 78.16 | 15 ; 20 | 3.34 |
| Acetoin | 89.05 | 71.00 ; 43.00 | 10 ; 40 | 6.51 |
| Acetylcholine | 147.11 | 87.98 ; 43.10 | 15 ; 20 | 2.54 |
| Adenosine | 268.09 | 136.02 ; 119.12 | 15 ; 30 | 4.13 |
| Adenosine diphosphate ribose | 560.07 | 136.13 ; 347.94 | 30 ; 15 | 11.97 |
| Agmatine sulfate | 229.08 | 72.09 ; 60.08 | 15 ; 15 | 9.52 |
| Aicar | 259.10 | 110.00 ; 127.10 | 24 ; 8 | 4.78 |
| Allose | 203.05 | 203.05 | 5 | 4.97 |
| Aminoadipate | 162.06 | 98.09 ; 116.08 | 15 ; 15 | 8.45 |
| Anserine | 241.12 | 108.97 ; 170.01 | 20 ; 15 | 10.68 |
| Arginine | 175.11 | 69.98 ; 60.09 | 15 ; 15 | 10.40 |
| Asparagine | 133.05 | 73.99 ; 87.22 | 15 ; 20 | 9.10 |
| Beta-alanine | 90.04 | 30.14 ; 72.01 | 15 ; 15 | 7.73 |
| Beta-NAD | 664.10 | 136.00 ; 428.00 | 40 ; 25 | 11.47 |
| Betaine | 118.07 | 59.09 ; 58.11 | 15 ; 15 | 6.49 |
| Biliverdin | 583.24 | 297.03 ; 299.05 | 30 ; 30 | 1.67 |
| Biotin | 245.08 | 227.00 ; 97.07 | 12 ; 30 | 2.05 |
| Cadaverine | 103.11 | 86.17 ; 69.12 | 15 ; 15 | 9.79 |
| Carnosine | 227.10 | 110.18 ; 155.94 | 15 ; 15 | 10.68 |
| CDP-Ethanolamine | 447.06 | 112.05 | 10 | 11.83 |
| Choline | 105.10 | 61.09 ; 45.14 | 15 ; 20 | 4.33 |
| Cis-4-Hydroxy-D-proline | 132.05 | 86.09 ; 68.12 | 15 ; 15 | 8.11 |
| Citicoline | 489.11 | 184.00 ; 263.90 | 30 ; 20 | 11.85 |
| Citrulline | 176.09 | 70.28 ; 112.82 | 20 ; 15 | 9.30 |
| Creatine | 132.06 | 90.06 ; 44.03 | 15 ; 20 | 7.96 |
| Creatinine | 114.05 | 44.17 ; 86.09 | 15 ; 15 | 4.06 |
| Cyclic GMP | 346.04 | 151.93 ; 134.98 | 20 ; 30 | 8.41 |
| Cystathionine | 223.06 | 87.90 | 32 | 11.40 |
| Cystine | 241.02 | 151.74 ; 74.27 | 15 ; 30 | 11.60 |
| Cytidine | 244.08 | 112.02 ; 95.24 | 20 ; 30 | 5.91 |
| Cytidine 2',3'-Cyclic phosphate | 306.04 | 112.16 ; 178.02 | 15 ; 15 | 8.54 |
| Cytosine | 112.04 | 95.09 ; 69.02 | 15 ; 15 | 4.99 |
| Alanine | 90.04 | 44.1 | 12 | 8.09 |
| Ornithine | 133.08 | 70.02 ; 116.03 | 15 ; 15 | 10.77 |
| Deoxyadenosine | 252.10 | 136.07 ; 117.09 | 15 ; 15 | 3.40 |
| Deoxyadenosine monophosphate | 332.06 | 136.01 ; 81.05 | 15 ; 20 | 9.68 |
| Deoxycarnitine | 146.11 | 87.16 ; 60.12 | 15 ; 15 | 5.99 |
| Deoxyguanosine | 268.09 | 152.00 ; 134.96 | 9 ; 30 | 5.58 |
| Deoxyuridine | 229.07 | 113.00 ; 69.06 | 10 ; 20 | 2.52 |
| Dethiobiotin | 215.13 | 197.09 ; 179.08 | 15 ; 20 | 1.66 |
| Diaminopimelate | 191.09 | 128.23 ; 82.16 | 15 ; 20 | 11.50 |
| Diethanolamine | 106.07 | 69.85 ; 44.95 | 20 ; 20 | 6.50 |
| Dihydrobiopterin | 240.10 | 168.06 ; 196.31 | 15 ; 15 | 3.15 |
| Dihydrouracil | 115.04 | 55.00 ; 44.00 | 20 ; 40 | 1.07 |
| Dopamine | 154.07 | 137.09 ; 91.14 | 15 ; 20 | 5.60 |
| DTDP-D-glucose | 565.07 | 587.07 | 5 | 11.63 |
| Epinephrine | 184.08 | 166.05 ; 107.14 | 15 ; 20 | 6.28 |
| FAD | 786.16 | 347.87 ; 135.83 | 20 ; 30 | 11.18 |
| Fumarate | 117.01 | 114.93 ; 91.03 | 15 ; 20 | 4.08 |
| Galactitol | 205.06 | 205.06 | 5 | 7.26 |
| Gamma-aminobutyrate | 104.06 | 87.17 ; 69.03 | 15 ; 15 | 6.92 |
| Glucosaminate | 196.07 | 72.03 | 20 | 12.40 |
| Glucosamine | 180.07 | 72.03 ; 162.26 | 15 ; 15 | 9.54 |
| Glutamate | 148.05 | 84.00 | 15 | 8.90 |
| Glutamine | 147.06 | 130.04 ; 84.00 | 15 ; 20 | 8.90 |
| Glutarylcarnitine | 276.14 | 85.01 ; 115.10 | 24 ; 19 | 5.45 |
| Glycine | 76.03 | 29.98 | 5 | 8.51 |
| Glycochenodeoxycholate | 472.61 | 472.61 | 5 | 2.58 |
| Glycocholate | 466.30 | 411.92 ; 430.07 | 15 ; 15 | 4.57 |
| Guanidinosuccinate | 176.05 | 88.03 ; 115.97 | 15 ; 15 | 8.80 |
| Guanine | 152.04 | 135.03 ; 110.13 | 20 ; 20 | 5.52 |
| Guanosine | 284.09 | 151.99 ; 135.02 | 20 ; 30 | 6.36 |
| Histamine | 112.07 | 95.22 ; 68.15 | 15 ; 20 | 9.13 |
| Histidine | 156.06 | 110.11 ; 82.99 | 15 ; 20 | 10.60 |
| Histidinol | 142.09 | 81.07 ; 124.12 | 15 ; 15 | 9.38 |
| Homocysteine | 136.03 | 56.00 ; 89.90 | 20 ; 8 | 7.98 |
| Homocystine | 269.05 | 136.00 ; 88.00 | 8 ; 40 | 10.69 |
| Hypoxanthine | 137.04 | 110.07 ; 119.08 | 20 ; 20 | 3.88 |
| Hydroxykynurenine | 225.08 | 162.00 ; 110.00 | 10 ; 20 | 12.10 |
| Inosine | 269.08 | 136.96 ; 119.20 | 15 ; 30 | 5.15 |
| Isoleucine | 132.09 | 69.14 ; 86.18 | 15 ; 15 | 6.54 |
| Kynurenate | 190.04 | 144.02 ; 172.03 | 20 ; 15 | 5.28 |
| Kynurenine | 209.08 | 93.91 ; 146.16 | 15 ; 15 | 6.27 |
| Carnitine | 162.10 | 60.08 ; 84.98 | 45 ; 45 | 7.04 |
| L-DOPA | 215.10 | 152.10 | 10 | 4.04 |
| L-Tryptophanamide | 204.10 | 159.10 ; 132.17 | 15 ; 20 | 5.37 |
| Lauroylcarnitine | 345.28 | 286.15 ; 84.97 | 15 ; 20 | 2.83 |
| Leucine | 132.09 | 86.15 ; 44.11 | 15 ; 20 | 6.29 |
| Lysine | 147.10 | 84.20 ; 129.96 | 15 ; 15 | 10.67 |
| Mannitol | 205.06 | 205.06 | 5 | 7.08 |
| Mannose | 203.05 | 203.05 | 5 | 4.97 |
| Methionine | 150.05 | 56.04 ; 104.01 | 15 ; 15 | 6.78 |
| Methylthioadenosine | 298.08 | 135.95 ; 119.00 | 15 ; 40 | 2.17 |
| Myoinositol | 203.05 | 203.05 | 5 | 4.97 |
| N-Acetylalanine | 132.05 | 43.98 ; 90.25 | 15 ; 15 | 1.89 |
| N-Acetylasparagine | 175.06 | 88.22 ; 157.87 | 15 ; 15 | 6.78 |
| N-Acetylcysteine | 164.03 | 164.03 ; 117.93 | 5 ; 15 | 6.28 |
| N-Acetylglutamate | 190.06 | 129.96 ; 84.00 | 15 ; 20 | 4.95 |
| N-Acetylleucine | 174.10 | 86.06 ; 132.08 | 15 ; 15 | 1.25 |
| N-Acetylneuraminate | 310.10 | 120.95 ; 273.96 | 20 ; 15 | 9.85 |
| N-Acetylphenylalanine | 208.08 | 120.06 ; 166.13 | 20 ; 15 | 1.35 |
| N-Acetylputrescine | 131.11 | 72.02 ; 114.11 | 15 ; 15 | 6.42 |
| N-Acetylserine | 148.05 | 130.02 ; 88.11 | 15 ; 15 | 5.16 |
| N-Alpha-acetyllysine | 189.11 | 84.09 ; 129.17 | 20 ; 15 | 8.30 |
| N-Formyl-L-methionine | 178.04 | 84.20 ; 104.04 | 20 ; 15 | 1.66 |
| N-Methylaspartate | 148.05 | 88.17 ; 42.03 | 15 ; 20 | 9.26 |
| N-Methylglutamate | 162.06 | 98.20 ; 116.04 | 15 ; 15 | 8.27 |
| N-Methyltryptamine | 175.11 | 144.10 ; 132.05 | 15 ; 15 | 3.23 |
| N,N-Dimethylarginine | 203.14 | 70.09 ; 88.01 | 20 ; 15 | 9.60 |
| N,N,N-Trimethyllysine | 189.15 | 84.09 ; 130.13 | 20 ; 15 | 10.12 |
| Nicotinamide | 123.04 | 80.06 ; 78.08 | 20 ; 20 | 1.40 |
| Nicotinate | 124.03 | 80.06 ; 78.02 | 20 ; 20 | 1.82 |
| Normetanephrine | 184.08 | 166.08 ; 134.25 | 15 ; 20 | 5.38 |
| O-Acetylserine | 148.05 | 88.17 ; 60,1 | 15 ; 20 | 7.15 |
| Ophthalmalate | 290.12 | 58.08 ; 160.99 | 20 ; 15 | 9.43 |
| Oxoproline | 130.04 | 84.14 ; 56.10 | 15 ; 28 | 5.20 |
| P-Octopamine | 154.07 | 91.06 ; 135.87 | 20 ; 15 | 5.58 |
| Palmitoylcarnitine | 401.34 | 84.89 ; 60.11 | 30 ; 20 | 2.55 |
| Pantothenate | 220.11 | 90.02 ; 71.96 | 15 ; 20 | 1.98 |
| Paraxanthine | 181.06 | 124.03 ; 69.00 | 20 ; 30 | 1.38 |
| Phenylalanine | 166.07 | 120.09 ; 103.13 | 20 ; 20 | 6.21 |
| Pipecolate | 130.07 | 56.15 ; 84.01 | 20 ; 15 | 7.27 |
| Proline | 116.06 | 70.11 ; 43.10 | 15 ; 30 | 7.13 |
| Purine | 121.04 | 94.11 ; 66.99 | 20 ; 20 | 1.95 |
| Putrescine | 89.10 | 71.95 ; 30.13 | 15 ; 15 | 10.11 |
| Pyridoxal | 168.05 | 150.10 ; 94.10 | 15 ; 20 | 2.12 |
| Pyridoxamine | 169.08 | 152.10 ; 134.01 | 15 ; 20 | 8.66 |
| Pyridoxine | 170.07 | 134.10 ; 152.02 | 40 ; 40 | 3.58 |
| S-Adenosylmethionine | 399.44 | 250.10 ; 136.00 | 28 ; 28 | 11.14 |
| Salsolinol | 180.09 | 180.09 ; 163.13 | 5 ; 15 | 5.21 |
| Sarcosine | 90.04 | 44.24 | 15 | 7.74 |
| Serine | 106.04 | 59.90 ; 42.00 | 15 ; 40 | 8.98 |
| Serotonin | 177.09 | 159.98 ; 131.91 | 15 ; 20 | 5.01 |
| Sorbitol | 205.06 | 205.06 | 5 | 7.08 |
| Taurine | 126.01 | 108.86 ; 64.85 | 15 ; 15 | 7.11 |
| Thiamine | 266.11 | 123.08 ; 145.06 | 15 ; 15 | 6.89 |
| Thymidine | 243.09 | 126.89 ; 117.13 | 15 ; 15 | 2.15 |
| Thymine | 127.04 | 83.88 ; 55.95 | 15 ; 15 | 1.50 |
| Thyrotropin releasing hormone | 363.17 | 115.12 ; 248.97 | 20 ; 20 | 7.90 |
| Trans-4-Hydroxy-L-proline | 132.05 | 86.03 ; 67.94 | 15 ; 15 | 8.12 |
| Trigonelline | 138.04 | 92.09 ; 94.11 | 20 ; 20 | 6.75 |
| Trimethylamine | 60.07 | 45.24 ; 44.10 | 15 ; 24 | 4.05 |
| Tryptamine | 161.10 | 144.10 ; 117.17 | 15 ; 20 | 3.98 |
| Tryptophan | 205.08 | 145.85 ; 188.19 | 15 ; 15 | 6.25 |
| Tyramine | 138.08 | 121.08 ; 77.08 | 15 ; 20 | 4.64 |
| Tyrosine | 182.07 | 136.23 ; 91.06 | 15 ; 30 | 7.19 |
| Uracil | 113.02 | 96.09 ; 40.30 | 15 ; 20 | 1.87 |
| Ureidopropionate | 133.05 | 72.02 ; 29.99 | 15 ; 15 | 3.00 |
| Uridine | 245.06 | 113.06 ; 70.00 | 15 ; 40 | 3.88 |
| Valine | 118.07 | 72.08 ; 55.00 | 15 ; 20 | 7.10 |
| Xanthine | 153.03 | 136.16 ; 109.87 | 15 ; 15 | 4.54 |
| Xanthosine | 285.07 | 152.98 ; 135.95 | 20 ; 30 | 5.62 |
| Xanthurenate | 206.03 | 160.08 ; 132.03 | 20 ; 30 | 5.66 |
| Acetylcarnitine | 204.10 | 85,10 ; 145.0 | 55 ; 12 | 5.72 |
| Propionylcarnitine | 218.20 | 85.10 | 19 | 4.88 |
| Butyrylcarnitine/Isobutyrylcarnitine | 232.15 | 85.10 ; 173.10 | 20 ; 13 | 4.36 |
| Tiglylcarnitine | 244.15 | 85.10 | 20 | 4.22 |
| Isovalerylcarnitine | 246.32 | 60.10 ; 85.10 | 17 ; 21 | 4.02 |
| Malonylcarnitine | 248.10 | 60.10 ; 85.10 | 18 ; 22 | 6.64 |
| 3-Hydroxybutyrylcarnitine | 248.10 | 189.10 ; 85.10 | 14 ; 22 | 6.64 |
| Hexenoylcarnitine | 258.20 | 85.10 ; 97.20 | 21 ; 17 | 3.90 |
| Hexanoylcarnitine | 260.15 | 85.00 ; 99.10 | 21 ; 17 | 3.74 |
| O-Succinyl-L-carnitine | 262.10 | 85.10 | 23 | 6.21 |
| 3-Hydroxyvalerylcarnitine | 262.20 | 85.10 ; 145.00 | 23 ; 16 | 6.21 |
| [(3R)-3-Hydroxyhexanoyl]-L-carnitine | 276.20 | 145.00 ; 85.10 | 16 ; 24 | 5.45 |
| 2-Octenoylcarnitine | 286.20 | 85.10 ; 227.10 | 22 ; 14 | 3.68 |
| Octanoylcarnitine | 288.20 | 85.10 | 22 | 3.34 |
| Adipoylcarnitine | 290.20 | 85.10 ; 213.10 | 26 ; 16 | 6.13 |
| Trans-2-Decenoylcarnitine | 314.20 | 85.10 ; 153.10 | 23 ; 18 | 3.15 |
| Decanoylcarnitine | 316.20 | 85.10 ; 155.10 | 23 ; 17 | 3.06 |
| Suberoylcarnitine | 318.20 | 85.10 ; 139.10 | 30 ; 22 | 5.21 |
| Trans-2-Dodecenoyl-L-carnitine | 342.30 | 85.10 ; 181.20 | 25 ; 18 | 2.87 |
| Sebacoyl-L-carnitine | 346.20 | 85.10 ; 139.10 | 31 ; 23 | 4.57 |
| [(3R)-3-Hydroxydodecanoyl]-L-carnitine | 360.30 | 85.10 ; 145.00 | 26 ; 19 | 4.04 |
| Cis,cis-5,8-Tetradecanedienoyl-L-carnitine | 368.30 | 85.10 ; 189.30 | 25 ; 18 | 2.73 |
| Trans-2-Tetradecenoyl-L-carnitine | 370.30 | 85.10 ; 209.20 | 25 ; 19 | 2.66 |
| Myristoylcarnitine | 372.30 | 85.10 ; 313.30 | 25 ; 16 | 2.66 |
| [(3R)-3-Hydroxytetradecanoyl]-L-carnitine | 388.30 | 85.10 ; 145.00 | 27 ; 20 | 3.89 |
| Trans-2-Hexadecenoyl-L-carnitine | 398.30 | 85.10 ; 237.20 | 27 ; 19 | 2.56 |
| Heptadecanoylcarnitine | 414.50 | 85.10 | 26 | 2.48 |
| [(3R)-3-Hydroxyhexadecanoyl]-L-carnitine | 416.30 | 85.10 ; 145.00 | 28 ; 21 | 3.75 |
| Cis,cis-9,12-Octadecadienoyl-L-carnitine | 424.60 | 85.10 | 20 | 2.49 |
| Oleoyl-L-carnitine | 426.40 | 85.10 | 27 | 2.44 |
| Stearoylcarnitine | 428.00 | 85.10 ; 267.30 | 27 ; 20 | 2.41 |
| [(3R)-3-Hydroxyoctadecanoyl]-L-carnitine | 444.40 | 85.10 ; 145.00 | 29 ; 22 | 3.63 |
| Arachidonyl-L-carnitine | 448.30 | 60.10 ; 85.10 | 23 ; 29 | 2.40 |
| Hypotaurine | 110.03 | 30 ; 92 | 15 ; 5 | 8.01 |
